# Report on Prostitution and Syphilis

**Published:** 1856-04

**Authors:** 


*Report on Prostitution and Syphilis.* — We give place to the following official document on a highly important subject, and containing matters of grave public interest, if the public health and morals of this vast population, perennially found in New York, be worthy of the regard of philanthropists and Christians. The report is ably drawn up, in sufficiently intelligible language, nor is there any exaggeration in its appalling details. Would that we could hope for its statements and inferences a just appreciation by the authorities of this corrupt and demoralized city.—*Am. Med. Gazette.*

*To Isaac Townsend, Esq., President of the Board of Governors of Alms House:*

In answer to your inquiries the Medical Board of Bellevue Hospital respectfully reply:

That they caused a census of the Hospital to be taken on the 24th October last, for the purpose of ascertaining what proportion of the patients had suffered from venereal diseases. From that enumeration they learn that out of 447 persons then under medical and surgical treatment, 142, or about one-third have been so affected. In the several divisions of the house the numbers are as follows:

Of 72 females on the surgical side, 17, or 1 in 4.24.

Of 130 females on the medical side, 17, or 1 in 8 nearly.

Of 118 male patients on the medical side, 45, or 1 in 2.6.

Of 127 males on the surgical side, 63, or 1 in 2.

So that out of 245 males then under treatment, 108, or 1 in 2.27, had had some form of venereal disease; and among 202 females, 34, or 1 in 6, had been similarly affected.

Of the whole number who confessed that they had had affections of this class, 106 had the Syphilis, and 36 had had Gonorrhœa.

Of 106 who had had Syphilis, 53, or just one-half were still laboring under the influence of the poison with which they had been inoculated, in many instances, years before.

As almost all of these patients were admitted for other diseases, or with affections which the physician alone would recognise as the remote effects of Syphilis, it is perhaps fair to assume that they represent, with some exaggeration, the class of society from which they come.

The Board has been favored with the census of the New York Hospital, (Broadway,) taken for the purpose of ascertaining the proportion of Syphilitic cases among the patients of that Institution; from which it appears that the whole number of patients on the 8th of December was 233, and that 99 of that number had had venereal disease, and 37 were then under treatment for the same affections, recently contracted. Counting the old cases alone, most of which were admitted, probably, for other diseases, this proportion exceeds that above recorded for Bellevue Hospital; it being as high as 1 in 2.35, It is proper, however, in this connection, to state, that the returns for Bellevue Hospital are believed to be incomplete. They are based in a considerable degree on the confessions of the patients; and it is known that many, especially among the women, have denied any contamination, when facts, subsequently developed, have shown that their statements were not true.

Is it to be believed, then, that one in three, or even one in four, of that large class of our population whose circumstances compel them to seek the occasional aid of medical charities, are tainted with venereal poison? This the Medical Board do not think they are authorized to state. But the facts here cited, and others within their reach, justify them in saying, that venereal diseases prevail to an alarming extent among the poor of this city. The large number of women sent by the Police Courts, to be treated for these diseases at the Penitentiary Hospital, would alone be sufficient evidence of this. Yet such persons constitute but a small proportion of those who, even among the poor, suffer from these disorders. Dispensary Physicians and those in private practice can show a much longer list of the victims of impure intercourse.

But the disease is not confined to this class. The advertisements which crowd the newspapers, introduced by men who "confine their practice to one class of disease, in which" they "have treated twenty thousand cases," more or less, demonstrates how large is the company of irregulars who live and grow on the harvest of these grapes of Sodom. And yet their long list of "unfortunates" would disclose but a fraction of the evil among those who are able to pay for medical services. The Medical Board are unable to state what proportion of the income of regular and qualified physicians in this city is derived from the treatment of venereal diseases, but they know it is large, and that many, who never advertise their skill, receive more from this source than from all other sources together. They believe that there is no one among the unavoidable diseases, however prevalent, for the treatment of which the well-to-do citizens of New York pay one-half so much as they pay to be relieved from the consequences of their illicit pleasures.

The city bills of mortality give little information regarding the frequency of venereal affections. *Lues venerea* keeps its place in the tables, and counts its score or two of deaths annually. Although this class of disorders is not frequently fatal, except among children, it is credited with only a fraction of the work it actually performs. The physician does not feel called upon, in his return of the causes of death, to brand his patient's memory with disgrace, or to record an accusation against near relatives. During infancy the real disease is buried under such terms as Marasmus, Atrophia, Infantile Debility, or Inflammation; while in adults, Inflammation of the Throat, Phagedæna, Ulceration, Scrofula, and the like, take the responsibility of the death.

These affections are strictly what the advertisers denominate them, "private diseases,"—a leprosy which "the unfortunate" always strives to conceal, and, so long as it spares his speech and countenance, usually succeeds in concealing. The physician is his only confident—and the physician refers all to the class of "innocent secrets," which are not to be revealed. The public, therefore, know little of the prevalence of such diseases, and still less of the fearful ravages they are capable of making.

Still, as has been just said, Syphilis is not often the immediate cause of death in adults. After its first local effects are over, (and these, though generally mild, are sometimes frightful,) the poison lingers in the system ready to break out on any provocation in some one of its many disgusting manifestations; often deforming all and branding its victim, threatening life and making it a burthen, and yet refusing the poor consolation of a grave. Like the vulture which fed on the entrails of the too amorous Tityus, it tortures and consumes, but is slow to destroy; and often its visible brand, like the scarlet badge once worn by the adulteress, proclaims a lasting disgrace. The protracted suffering of mind and body produced by this class of distempers, the ever-changing and often loathsome forms of their secondary accidents, and the almost irradicable character of the poison, seem almost to justify an old opinion, sanctioned by a Papal Bull as lately as 1826, that these diseases are an avenging plague, appointed by Heaven as a special punishment for a special sin.

The relentless character of Syphilitic disease stands out in painful relief in its transmission from parent to offspring. Here it is, indeed, that the children's teeth are set on edge, because the fathers have eaten sour grapes. The contaminated husband or wife is left through years of childlessness, or of successive bereavements, to mourn over early follies, and to repent when repentance is fruitless. The Syphilitic man or woman can hardly become the parent of a healthy child.

A young man has imbibed the contagion — it has become constitutional. After a few weeks, or months perhaps, of treatment, the visible signs of the disease no longer torment him. He has contracted a matrimonial alliance, and soon marries a healthy and virtuous woman. He flatters himself that he is cured. A few months suffice to give him painful proofs of his error, for then his growing hopes of paternity are suddenly blasted. Instead of the child of his hopes, he sees a shrive ed and leprous corpse. This is but the first in a series of similar misfortunes. He has poisoi ed the fruit of his loins, and again and again, and still it falls withered and dead. At length nature seems to have triumphed over this foe to domestic happiness, and the parents' hearts are gladdened by the prospect of a living child. Their joy is short-lived. The child is feeble and sickly, and in a few days or weeks another death is added to the penance-list of the humbled and grieving father.

This mournful story will need no essential change in the narration, should the poison of impure intercourse, legitimate cr illicit, linger in the veins of the mother.

A child of such a connection may be born in apparent health, but before six months have passed, some one of the numerous forms of infantile Syphilis will be likely to appear and threaten its life. In the contest which follows between disease and the treatment, the physician is commonly victorious, but the contest is in many cases protracted, and often is it to be renewed again and again. And after all, it is not believed that children thus tainted at their birth often grow up and acquire that degree of health and vigor which is popularly ascribed to a *good constitution.*

These are facts familiar to physicians practising in large towns. But the history of inherited Syphilis is not yet complete. If, in the case just recited, the wife escape contamination from her husband and from her unborn child, yet the sad consequences of that husband's folly are not yet exhausted. The tainted child, now a sickly nursling at her breast, has a venom in its ulcerated lips and throat, which can inoculate the mother with its own loathsome poison, while it draws its sustenance from the sacred fountain of infantile life. But this is not all. These little innocents sometimes spread their disease through the whole circle of those who bestow on them their care and kindness. The utmost caution and cleanliness cannot always save those who dress their poisonous sores. The contagion spreads sometimes through the use of the same spoon, the same linen, and even by that highest token of affection, a kiss. It has been known that a single diseased child has contaminated its mother; a hired nurse; and through that nurse, the nurse's child; and in addition to these, the husband's mother, and the mother's sister. Such are sometimes the weighty consequences of a single error.

PREVENTION.

That the great source of the venereal poisons is prostitution, requires no argument. The first question then to be answered is — Can prostitution be prevented? In answering this question, it is necessary to remember that the history of the world demonstrates the existence of this vice in all ages, and among all nations, since the day its first pages were written. The appetite which incites has always been stronger than moral restraints—stronger than the law. No rigor of punishment, no violence of public denunciation; neither exile, nor the dungeon, nor yet the disgusting malady with which nature punishes the practice, has ever effected its extermination, even for a single year. Great as this evil has always been, it cannot be denied that, in our own time, some of the accidents of what is called *the progress of society* tend, at least in large towns, greatly to increase it. The expenses of living are everywhere the great obstacle to early marriages; whether such expenses be positively necessary, or be demanded by the social position of the individual — the fashion of his class — and therefore become relatively necessary. Whenever these expenses increase more rapidly than the rewards of labor, marriage becomes impossible for a constantly increasing number, or can only be purchased at the price of social position. But abstinence from marriage does not abolish or moderate the natural appetites. The great law of nature on which the existence of the race depends is not abrogated by any artificial state of society. Moral or religious principles will restrain its operation in some; human laws in some; the fear of consequences in some; yet there always have been, and probably always will be, many of both sexes who are not restrained by any of these considerations. These have sustained, and probably will continue to sustain, not only prostitution, but houses of prostitution, in the face of every human law. Suppressed in one form, it immediately assumes another. Again pursued, it retreats to hiding places where darkness and secrecy protect it from the pursuer.

Severe penalties have heretofore only increased the evils of prostitution. If a hundred women are consigned to prison for this vice to-day, before a month has elapsed as many more have taken their places; and the hundred, though punished, are not reformed. Impelled by a love of their profession, or some by the passion to emulate the more fortunate of their sex in the finery of dress, (a passion which perhaps first occasioned their fall,) many by want, and all by a sense that they are outcasts, they are no sooner liberated than they return with new zeal to the life from which they have been detained only by force. Severe laws compel secrecy — they can do no more. When prostitution is criminal, disease, if known to others, is a practical conviction. Under such circumstances, the contaminated will be slow to confess disease, and so subject themselves to punishment. Yet their passion and their necessities alike forbid even temporary abstinence. They spread disease without limit.

Under this fact lies an important thought — Were it no more disgraceful to contract Syphilis than it is to have fever and ague, the diseased would seek early relief, which is nearly equivalent to certain relief, and the disorder would soon be confined to the pitiable few, who have lost in drunkenness and misery the instinctive dread of all that is foul and disgusting in personal disease. Prostitution, it is true, would then be restored to its old Roman dignity, yet venereal disease could then be reached, and all but eradicated. But a *respectable Syphilis* does not belong to our age and nation. It lost caste in the beginning, and its exploits in modern times have not been of a character to win it friends. The supposition aims only to show, by contrast, he evils of well-intended, but probably unjudicious legislation. Regarding pains and penalties: if the whip, confiscation, and banishment, in the hands of Charlemagne and St. Louis, aided by a right good will, and all the powers of a military despotism, could not suppress prostitution, or even prevent the opening of houses of prostitution; if penal laws in Europe, from the days of these earnest princes until now, have utterly failed of their object, as they notoriously have, it is fair to ask, how much more can prohibitory laws accomplish in a country where the right of private judgment, and personal liberty in speech and action, are the very foundation of the body politic? They have hitherto been ineffectual. In spite of such laws the vice is increasing. *In consequence of such laws its most enormous physical evil is extending its baleful influence through every rank and circle of society.* It is still emphatically the plague of the poor — it still brings sorrow and misery to the fire-sides of the affluent and titled.

An utopian view of the perfectability of man might look for the remedy to this evil in universal early marriages, in domestic happiness, and in a universal moral sense, which will compel men and women to keep their marriage vows. But taking man as he is, we find the tides of society set with constantly increasing strength against early marriages; that domestic happines is not synonymous with marriage, whether early or late; and that the moral sense which should teach all men to observe even their solemn promises, would be miraculous. For these things the law has done all that has been thought wise to attempt, probably all that it can do.

But it may be asked, if government has the power to relieve society of the vice of drunkenness, why despair of its power regarding prostitution? In reply it may be asked, if the drunkard himself is ever cured of his vicious appetite by penalties? The statute despairs of this. It even recognises its inability to prevent the sale of intoxicating drinks while they exist; it therefore claims the right to seize and destroy them. Can it seize on and destroy the inborn passion which fills and supports houses of prostitution? Then it cannot do for the one what it hopes to do for the other.

Again, the suppression of slavery and the slave trade have been cited in connection as illustrating the power of law. But these offences have no hiding places; they cannot assume the guise of virtue and still be vice; they are patent as the light, darkness and secrecy cannot cover them.

☞ Prostitution is unlike any offence that is within the grasp of the law. In tresspass, theft, or violence, or fraud, some one is wronged; and those who have been injured seek to bring the offender to justice. Here there is no aggrieved person. All who are in interest, are so in interest that they deprecate the interference of all law, except what they claim to believe is law of nature.

But is there no hope in the Societies of Moral Reform? For the suppression or even checking of general vice, none whatever. The Association in New York deserves much praise for its zealous benevolence. They have brought back some of those erring women to the paths of virtue, but they have done no more to stop the current of prostitution, than he could do to dry up the Hudson who dips water with a bucket. In truth it may be said, that the paths of virtue have been found to be slippery places for some that would be thought converts. Wisdom's ways have been found to be too peaceful for these daughters of excitement. This is said in no spirit of disparagement to the efforts of the Society. They may well be proud of what they have done. But it is said to show how little the kindest and best can do to reclaim those who have once fallen from virtue and honor.

Let the great fact, then, be well understood, that prohibitory measures have always failed, and from the nature of the case must forever fail to suppress prostitution.

Let this additional fact, illustrated in the foregoing remark, be well considered; that penalties do not reform the offender, but that they enforce secrecy in the offence, and silence regarding its consequences, which is a chief cause of the present wide diffusion of the venereal poison.

What then is the proper province of legislation in this important matter?

The wise law-giver does not attempt impossibilities. He knows that laws which experience has demonstrated cannot be enforced, teach disrespect and disobedience to all law. He knows that human passions cannot be changed by human legislation. He knows that if he attempt the impossible greater, in the control of vice, he is certain to neglect the possible and important less. He knows that the river will not cease to flow at his command. If it overflows and desolates, he raises its banks and dykes in the flood to prevent a general inundation. For hundreds of years the governments of Europe have tried in vain to dry up the sources of prostitution; with the opening of the present century, they began to dyke in the river and prevent avoidable mischief. For a long time we too have had laws against prostitution, which, with every proper effort by those in authority, have proved as useless as those who live by this illicit traffic could desire, as mischievous in spreading disease as the quack advertiser could wish. Is it not time then to inquire whether we have not attempted too much, whether if we attempt less we shall not accomplish more? May we not be able to limit and control what we have not the power to prevent? If we cannot do all that a large benevolence might wish to accomplish, in the name of humanity, is not our duty to do what is useful and practicable—all that is possible?

While the Medical Board are persuaded that by a change of policy, such as is suggested by the facts and reasons herewith submitted, much can be done to limit and control prostitution, and much more toward the eradication of venereal diseases, they are not yet prepared to offer the details of a plan by which they hope these important ends can be attained. With the assistance of the Board of Governors, they are now in correspondence with the Medical officers of many of the larger cities of Europe, where restrictive measures have replaced prohibitory. When they have obtained the information which they hope this correspondence will furnish, they will ask leave to submit a supplementary Report.

JOHN W. FRANCIS, M. D., *President.*

JNO. T. METCALFE, M. D., *Secretary pro tem.*

